# Genome-Wide Association and Gene Co-expression Network Analyses Reveal Complex Genetics of Resistance to Goss’s Wilt of Maize

**DOI:** 10.1534/g3.119.400347

**Published:** 2019-07-30

**Authors:** Amritpal Singh, Guangyong Li, Alex B. Brohammer, Diego Jarquin, Candice N. Hirsch, James R. Alfano, Aaron J. Lorenz

**Affiliations:** * Department of Agronomy and Plant Genetics, University of Minnesota, St. Paul, MN 55108; † Department of Plant Pathology, University of Nebraska, Lincoln, NE 68503-0722; ‡ Center for Plant Science Innovation, University of Nebraska, Lincoln, NE 68588-0660, and; § Department of Agronomy and Horticulture, University of Nebraska, Lincoln, NE 68503-0915

**Keywords:** Maize (*Zea mays*), genome-wide association mapping, quantitative disease resistance, Goss’s wilt, weighted gene co-expression network analysis, Clavibacter, quantitative trait loci, genotyping-by-sequencing, single nucleotide polymorphism

## Abstract

Goss’s bacterial wilt and leaf blight is a disease of maize caused by the gram positive bacterium *Clavibacter michiganensis* subsp. *nebraskensis* (*Cmn*). First discovered in Nebraska, Goss’s wilt has now spread to major maize growing states in the United States and three provinces in Canada. Previous studies conducted using elite maize inbred lines and their hybrids have shown that resistance to Goss’s wilt is a quantitative trait. The objective of this study was to further our understanding of the genetic basis of resistance to Goss’s wilt by using a combined approach of genome-wide association mapping and gene co-expression network analysis. Genome-wide association analysis was accomplished using a diversity panel consisting of 555 maize inbred lines and a set of 450 recombinant inbred lines (RILs) from three bi-parental mapping populations, providing the most comprehensive screening of Goss’s wilt resistance to date. Three SNPs in the diversity panel and 10 SNPs in the combined dataset, including the diversity panel and RILs, were found to be significantly associated with Goss’s wilt resistance. Each significant SNP explained 1–5% of the phenotypic variation for Goss’s wilt (total of 8–11%). To augment the results of genome-wide association mapping and help identify candidate genes, a time course RNA sequencing experiment was conducted using resistant (N551) and susceptible (B14A) maize inbred lines. Gene co-expression network analysis of this time course experiment identified one module of 141 correlated genes that showed differential regulation in response to *Cmn* inoculations in both resistant and susceptible lines. SNPs inside and flanking these genes explained 13.3% of the phenotypic variation. Among 1,000 random samples of genes, only 8% of samples explained more phenotypic variance for Goss’s wilt resistance than those implicated by the co-expression network analysis. While a statistically significant enrichment was not observed (*P* < 0.05), these results suggest a possible role for these genes in quantitative resistance at the field level and warrant more research on combining gene co-expression network analysis with quantitative genetic analyses to dissect complex disease resistance traits. The results of the GWAS and co-expression analysis both support the complex nature of resistance to this important disease of maize.

Goss’s bacterial wilt and leaf blight of maize, caused by a gram positive bacterium *Clavibacter michiganensis* subsp. *nebraskensis* (*Cmn*), can dramatically reduce grain yield of maize if infection becomes severe enough. Studies using artificial inoculations of *Cmn* conducted during the 1990s showed yield losses up to 44% (Carson and Wicks 1991), and yield losses of 50% or more have been reported by producers (Robertson 2012). Goss’ wilt was first discovered on one farm in southcentral Nebraska in 1969 (Schuster *et al.* 1972). Over the next decade, Goss’s wilt spread to 58 counties in Nebraska and 34 counties across seven states, becoming a major concern for maize producers (Wysong *et al.* 1981). The incorporation of host plant resistance into maize hybrids greatly reduced the incidence of Goss’s wilt, with it being only sporadically observed in the far western Corn Belt throughout the late 1980s until the mid-2000s (Jackson *et al.* 2007). Goss’s wilt suddenly re-emerged around 2006 as indicated by a large increase in the number of samples diagnosed with Goss’s wilt submitted to plant disease diagnostic clinics (Jackson *et al.* 2007). Since 2006, Goss’s wilt has continued to spread throughout North American maize growing regions, having been reported in Nebraska, Iowa, Colorado, Missouri, Indiana, Illinois, Kansas, Minnesota, North Dakota, South Dakota, Wisconsin, Texas, and Louisiana. In Canada it has been confirmed in Alberta, Manitoba, and Ontario (Ruhl *et al.* 2009; Malvick *et al.* 2010; Korus *et al.* 2011; EPPO 2014; Friskop *et al.* 2014; Howard *et al.* 2015; Singh *et al.* 2015; Hosack *et al.* 2016). In recent years Goss’s wilt has ranked among major diseases of maize in North America in terms of estimated yield losses, with estimates ranging from 38.5 million bushels in 2012 to 215.9 million bushels in 2014 in major maize producing U.S. states and Canada (Mueller and Wise 2012, 2014; Mueller *et al.* 2016).

Identifying sources of resistance to Goss’s wilt and breeding resistance into maize hybrids remains a viable strategy to reduce yield losses. A great degree of variation in the level of resistance to Goss’s wilt exists in maize. Maize inbred lines have shown variable response to Goss’s wilt varying from resistant, intermediate, to highly susceptible based on screenings in the 1970s and 1980s with a limited number of inbred lines (Schuster *et al.* 1972; Calub *et al.* 1974; Wysong *et al.* 1981, Treat *et al.* 1990). For example, B14 and its derived lines, such as A619, have been found to be generally susceptible; Oh43 was reported to be moderately susceptible; and Mo17 was reported to be resistant (Schuster *et al.* 1972; Calub *et al.* 1974). Studies using classical mating designs, including diallels and generation means analyses, have indicated that resistance to Goss’s wilt is under polygenic control, with additive genetic variation being the predominant form of genetic variation (Gardner and Schuster 1974; Martin *et al.* 1975; Rocheford *et al.* 1989; Treat and Tracy 1990; Ngong-Nassah *et al.* 1992).

Efforts to identify molecular markers linked to resistance to help unravel the genetic architecture of Goss’s wilt have been lacking until recently because the incidence of this disease was only sporadic during the time plant geneticists and breeders were developing and adopting molecular markers, QTL mapping, and marker-assisted selection (circa late 1980s to 2000s). The re-emergence of Goss’s wilt has increased interest in identifying sources of resistance, molecular markers that could be used for selection, and genes controlling resistance. Using three bi-parental maize populations connected by a common parent, Singh *et al.* (2016) identified eleven QTL of small effect, half of which were population specific. Schaefer and Bernardo (2013) performed genome-wide association mapping for Goss’s wilt and identified several SNPs associated with resistance, each explaining no more than 10% of the phenotypic variation. The diversity panel used by Schaefer and Bernardo (2013), however, was limited in size and diversity, being comprised of only elite maize inbred lines. This limited diversity, combined with relatively few molecular markers, lowered the mapping resolution achieved by this study. Most recently, Cooper *et al.* (2018) used the Intermated B73 X Mo17 mapping population and related introgression lines to map QTL controlling Goss’s wilt resistance. These authors detected several of the previously mapped QTL, including a QTL on chromosome 1 that overlapped with a locus known to be important in resistance to multiple diseases of maize (Cooper *et al.* 2018).

Transcriptomic approaches, such as RNA-seq, can help identify resistance candidate genes by quantifying changes in gene expression levels after resistance and susceptible plants have been exposed to a pathogen. Several studies, however most of them involving fungal pathogens of maize, have investigated the response of maize to its pathogens using transcriptomics (Lanubile *et al.* 2014; Liu *et al.* 2015, 2016; Miranda *et al.* 2017). From these studies, several genes have been reported to be differentially expressed in resistant and susceptible lines in response to *Fusarium* and *Bipolaris* species with enrichment in functions including pathogen recognition and signaling, growth and development, plant hormone signal transduction, and defense (Liu *et al.* 2016; Lanubile *et al.* 2014; Liu *et al.* 2015). Study of gene expression changes in near-isogenic lines for disease resistance QTL to *Fusarium* further revealed two different mechanism of resistance provided by the QTL, where one QTL conferred resistance through the expression of defense related genes and the other imparted resistance by affecting auxin signaling and transport (Liu *et al.* 2016). Systemic acquired resistance in maize to *Colletotrichum* was found to be controlled by genes involved in salicylic acid pathway and chromatin modification (Miranda *et al.* 2017).

Recently published transcription profiling of resistant and susceptible maize genotypes (Doehlemann *et al.* 2008; Meyer *et al.* 2017; Yu *et al.* 2018), expression QTL analysis (Christie *et al.* 2017), and meta-analysis of expression profiling studies (Wang *et al.* 2018) for *Cercospora zeina*, *C. zeae-maydis*, and *Ustilago maydis* pathogens also revealed complex defense response and molecular interaction with maize. Different approaches revealed changes in hormonal signaling such as jasmonic acid, and salicylic acid, as well as oxidative reduction in response to pathogens. Studies also pointed toward implication of genes involved in primary defense such as leucine rich repeat receptor like kinases, PAMPs, flavonoid and terpenoid biosynthesis genes, calmodulin-like proteins, and genes encoding pathogenesis related proteins.

Combing GWAS and transcriptomic analyses provides a powerful systems approach to characterize the genetic basis of resistance to this pathogen of maize. The objectives of this study were: 1) Characterize the genetic variation for Goss’s wilt within a large panel of diverse inbred lines; 2) Discover genomic regions and candidate genes controlling variation for resistance to Goss’s wilt; 3) Discover co-expressed modules of genes that show changes in gene expression patterns between a resistant and susceptible maize line in response to inoculation with *Cmn*, and compare these genes to those implicated by GWAS; 4) Quantify the amount of variation in field resistance explained by differentially expressed gene modules.

## Materials and Methods

### Germplasm and selection of diversity panel for genome-wide association analysis

Two sets of germplasm were used for the GWAS: a diversity panel of 555 inbred lines and three bi-parental linkage mapping populations selected from the maize nested association mapping (NAM) population. The diversity panel of 555 maize inbred lines was selected from a larger set of 2,815 maize inbred lines genotyped by Romay *et al.* (2013). This panel consisted of lines from Stiff Stalk Synthetic (SSS), non-SSS, tropical, popcorn, and sweet corn genetic backgrounds. Subpopulation classification (*i.e.*, SSS, non-SSS, popcorn) of each line was according to Romay *et al.* (2013). The first year of Goss’s wilt resistance evaluation included 400 inbred lines selected from the total set of 2,815 inbred lines. To arrive at this set of 400, the initial set was first reduced to 900 lines by retaining only those that reached mid-silk within four days of B73 according to mid-silk growing degree data provided by Romay *et al.* (2013). This was done to reduce confounding effects that variation in days to flowering could have on disease ratings. A *k*-means clustering analysis was then applied to SNP data to classify the 900 lines into 400 clusters, and one line from each of the 400 clusters was randomly selected. Seed of the 400 inbred lines was obtained from the North Central Regional Plant Introduction Station (NCRPIS) in Ames, IA and increased during the summer of 2014 in the breeding nursery through self-pollination. Insufficient statistical power to detect associations in the first year of this study prompted an increase in the panel size for evaluations in 2015. The panel evaluated in 2015 consisted of 555 lines, which was formed by the addition of 155 inbred lines from the set of 900 for which adequate seed quantities were immediately available. No specific criteria were applied to selection of these additional 155 lines beyond immediate seed availability.

The three bi-parental RIL populations selected from the NAM population were B73 x Oh43, B73 x HP301, and B73 x P39. These same populations were used in an earlier study to identify QTL for Goss’s wilt through linkage mapping (Singh *et al.* 2016). While the common parent, B73, is moderately resistant, the other three parents were found to be comparatively susceptible to Goss’s wilt in a preliminary screening. Moreover, Oh43, HP301, and P39 are dent, popcorn, and sweetcorn types, respectively, which allowed for the discovery of Goss’s wilt resistance alleles from three distinct genetic backgrounds. In 2012, 195 RILs from the B73 x Oh43 population were screened for Goss’s wilt. In 2013, 172 RILs from the B73 x Oh43 population, 141 RILs from the B73 x HP301 population, and 125 RILs from the B73 x P39 population were evaluated. In 2014, 174 RILs from the B73 x Oh43 population, 143 RILs from the B73 x HP301 population, and 124 RILs from the B73 x P39 population were phenotyped for Goss’s wilt. The lines were evaluated using a completely randomized field design with replicated checks as described earlier (Singh *et al.* 2016).

### Genotypic data

ZeaGBSv1.0 dataset consisting of 681,257 GBS SNPs published by Romay *et al.* (2013) downloaded from panzea.org in 2013 was used to select the diversity panel. In 2014, ZeaGBSv2.7 version dataset of 955,690 GBS SNPs was made available for maize germplasm public germplasm including NAM RILs at panzea.org, which was then downloaded and used for the genome-wide association analysis. Briefly, the GBS data made available by Romay *et al.* (2013) was obtained using the *ApeKI* restriction enzyme as previously described (Elshire *et al.* 2011). A reference genome-based GBS pipeline in TASSEL 5.0 was used for SNP discovery with standard parameters as applied to maize Discovery Build was used to call the SNPs (Glaubitz *et al.* 2014). The imputed version of the ZeaGBSv2.7 dataset, imputed using Fast Inbred Line Library ImputatioN (FILLIN) method, was used (Swarts *et al.* 2014).

Markers with more than 80% missing data and a minor allele frequency (MAF) less than 0.05 were filtered out, leaving 342,237 SNPs for analysis. This filtered and imputed SNP dataset was used for principal component analysis (PCA), LD decay, and GWAS analyses.

### Diversity panel characterization

Population structure within the diversity panel was visually assessed using PCA and ADMIXTURE, a software for model-based estimation of ancestry (Alexander *et al.* 2009). A cross validation procedure implemented in ADMIXTURE was used to initially choose the optimum number of subpopulations (K) by minimizing the cross validation error. Multiple runs of ADMIXTURE were conducted at different values of K ranging from 3 to 20. Twenty replications were performed for each value of K. Cluster memberships for each replicate were aligned using the cluster matching software CLUMPP (Jakobsson and Rosenberg 2007). A plot of cross validation error *vs.* K was examined, but an exact optimum could not be determined (Supplementary Figure 1). K was set to three based on visual inspection of a PCA plot of PC1 *vs.* PC2 (Supplementary Figure 2a), subpopulation membership plots from ADMIXTURE differing in K (Supplementary Figure 2b), and subpopulation information from Romay *et al.* (2013). The average cluster membership across twenty replications of the ADMIXTURE analysis was used as a covariate in the GWAS model to account for population structure.

Decay of linkage disequilibrium (LD) was assessed as pairwise R^2^ between the SNP markers within 10 kb windows using PLINK v1.07 available at http://zzz.bwh.harvard.edu/plink/ (Purcell *et al.* 2007). To assess the relationship among the lines of the diversity panel and visualize the clustering of inbred lines according to Goss’s wilt resistance, a Hamming distance matrix was created using PLINK in which the distance was calculated as 1-IBS, where IBS is the identity-by-state coefficient. The distance matrix was used to create a neighbor joining tree using the method of Saitou and Nei (1987) as implemented in *ape* package in R (Paradis *et al.* 2004).

### Goss’s wilt phenotyping and disease nursery

In 2014, the diversity panel of 400 inbred lines was planted in a Goss’s wilt nursery at the Agricultural Research and Development Center of the University of Nebraska in Mead, NE. Plots were arranged in a randomized complete block design with three replications. Susceptible line B14A, and two susceptible and two resistant proprietary check inbred lines from Dow AgroSciences were included to assess disease development. In 2015, the diversity panel of 555 lines was planted at the same location using the same experimental design as in 2014. Inoculations with *Cmn* were carried out following the same procedure as described previously (Singh *et al.* 2016). Briefly, wounds were created on plant leaves with motorized weed whippers and *Cmn* inoculum was sprayed within seconds of injuring the plants to ensure infection. Disease ratings were recorded 15, 30, and 45 days after inoculations (DAI). A disease rating scale of 1 to 9 on a whole plot basis was used in this study, where 1 represents complete resistance, 2 indicates disease spread less than approximately 5 cm from the point of inoculation, 3 represents limited spread but more than 5 cm from the point of inoculation, 4 indicates a large spread with lesions often extending to middle of the leaf, 5 indicates systemic infection and lesions on un-inoculated leaves, 6 indicates blight of un-inoculated leaves and wilting of plants, 7 indicates severe blight and wilt, 8 indicate severe blight and severe wilt with limited green tissue on leaves and stems of plants, and 9 represents a completely dead plot (Singh *et al.* 2016).

### Analysis of phenotypic data

Three visual ratings taken after inoculation at 15, 30, and 45 DAI were combined to calculate WMD scores. For calculation of WMD, the average of two consecutive ratings was taken and multiplied by the number of days between the two ratings. These values were summed and divided by the total number of days spanning the first and last rating (Balint-Kurti *et al.* 2010; Singh *et al.* 2016). Analysis of variance (ANOVA) was conducted on WMD values using ASReml-R (Butler *et al.* 2009) by fitting the following model:

$$y_{ijk}=\mu +g_{i}+e_{j}+r_{k(j)}+ge_{ij}+\varepsilon_{ijk}$$ [1]

Where, $y_{ijk}$ represents the WMD value, μ is the grand mean, $g_{i}\;$is the effect of genotype *i*, $e_{j}$ is the effect for year *j*, $r_{k(j)}\;$is the effect of the *k*^th^ replication nested within the *j*^th^ year, $ge_{ij}\;$is the interaction effect between inbred line and year, and $\varepsilon_{ijk}\;$is the residual. All effects except for the residual were treated as fixed effects. Best linear unbiased estimates (BLUEs) for WMD of the inbred lines were calculated. For estimating heritability, inbred line, inbred line-by-year interaction, and residual variances were estimated using ASReml-R by fitting these effects as IID random effects. Plot-based heritability was calculated as $H^{2}=\frac{\sigma_{G}^{2}}{\sigma_{G}^{2}+\sigma_{GY}^{2}+\sigma_{\varepsilon }^{2}}\;$where $\sigma_{G}^{2}$ is the variance among inbred lines, $\sigma_{GY}^{2}\;$is the inbred-by-year interaction variance, and $\sigma_{\varepsilon }^{2}$ is the residual variance.

### Genome-wide association model

Genome-wide association mapping was performed using the following mixed linear model:

y=Xβ+wm+Zu+e [2]

where **y** is a vector of WMD BLUEs of the inbred lines; β is a vector of fixed effects, including an intercept and subpopulation (K = 3) effects; **X** is an incidence matrix relating β to **y** and contains subpopulation membership probabilities output from ADMIXTURE; *m* is the fixed SNP effect of the SNP being tested; **w** is a vector indicating the allelic state of each inbred line for the marker being tested; **u** is a vector of random polygenic effects where $u∼MVN(0,G\sigma_{u}^{2})$ and **G** is a genomic relationship matrix (GRM) calculated using the marker data; **Z** is a design matrix relating **u** to **y**; and **e** is a vector of random residuals where $e∼MVN(0,I\sigma_{e}^{2})$. Model [2] was implemented using the factored spectrally transformed linear mixed model (FaST-LMM) algorithm, including calculation of the GRM (Lippert *et al.* 2011). Parameter -fileSim was added to FaST-LMM command to obtain the GRM which uses a realized relationship matrix from SNPs to calculate the GRM.

The linear mixed model described was also applied to the combined dataset of diversity panel (N = 555) and bi-parental populations (N = 450). The model was modified to include a fixed environmental effect to account for the different environments in which these sets of germplasm were evaluated. Also, subpopulation effects were extended to include subpopulation effects for each of the three bi-parental populations. The subpopulation effect incidence matrix, *X*, which included subpopulation memberships as described above, was extended by adding three columns for each of the bi-parental populations, where each column contained a 1 when the RIL in that row belonged to the corresponding bi-parental population and a 0 in all other columns.

In order to declare SNPs as significantly associated with WMD in GWAS analyses, a false-discovery rate (FDR) based on a q-value of 0.1 was used (Storey and Tibshirani 2003). To calculate the percent variation explained by significant SNPs ($R_{SNP*}^{2}$) after accounting for subpopulation effects, a multiple regression model was fit including WMD BLUEs as the dependent variable, and subpopulation effects and those of significant SNPs as independent variables (full model). A model including only subpopulation effects as independent variables was also fit (reduced model), and $R_{SNP*}^{2}$ was calculated as the difference in variation explained between the full and reduced models.

The total variation in WMD BLUEs within the diversity panel that could be explained by all genotyped SNPs (polygenic background effects) was calculated using a variance component approach (Gusev *et al.* 2014):

$$\hat{g}=u+e$$ [3]

where $\hat{g}$ is the BLUE for inbred lines estimating using model [1], *u* is a random polygenic background effect where $u∼MVN(0,G\sigma_{u}^{2}$), and *e* is residual. The proportion of variation explained by all genotyped SNPs was calculated as σu2σg+2σe2 .

### Haplotype analysis

Once significant SNPs were identified by the GWAS, a haplotype analysis was performed in the regions of the genome surrounding the significant SNPs using the software Haploview (Barrett *et al.* 2005). The SNPs within 10 kb of each significant SNP were included initially to conduct the haplotype block analysis. The regions were extended beyond 10 kb for haplotype block analyses if the haplotype block length in a region was greater than 10 kb. Haplotype blocks were defined according to the four gamete rule (Wang *et al.* 2002). A cutoff of 1% was used to define the haplotype block boundaries, meaning that if a fourth two-SNP haplotype allele was observed at a frequency of greater than 1%, recombination was assumed to have occurred between the SNPs that formed the haplotype. Allele frequencies of haplotype alleles were examined within each subpopulation of the diversity panel to determine the allele frequency differences among subpopulations.

### Plant materials and inoculations for transcriptome profiling

**Figure 1:**
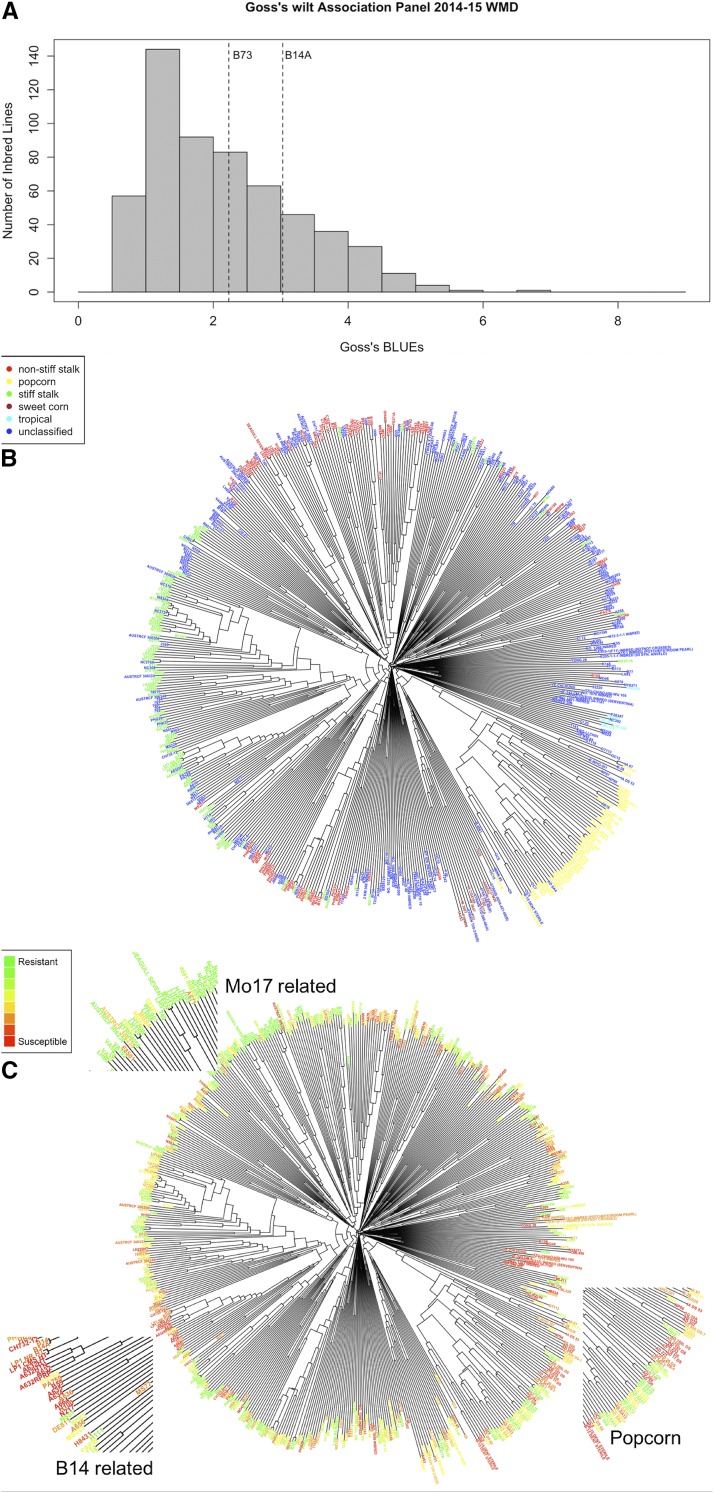
Genetic relationship among the inbred lines of the diversity panel and distribution of Goss’s wilt among the inbred lines. (a) Histogram showing the distribution of weighted mean disease BLUEs for inbred lines included in the diversity panel. Vertical dotted lines display the check lines B73 and B14A; (b) Neighbor joining tree of 555 lines of the diversity panel created from a distance matrix calculated using the GBS SNP data. Labels are colored coded by the six subpopulation groups within the diversity panel; (c) The same neighbor joining tree as in (b) but labels are color coded according to Goss’s weighted mean disease values. Red color indicates that a line is susceptible and green color represents resistant lines. Specific groups are zoomed to indicate trends in Goss’s wilt resistance distribution. For example, most of the B14-related lines were susceptible while Mo17-related lines were relatively resistant. N551 is present in the B14 zoomed window along with B14A.

Transcriptome profiles were obtained for B14A and N551 under both control and *Cmn* inoculated conditions. B14A was found to be susceptible and N551 was found to be resistant to *Cmn* in the field screening described above (Figure 1c). Mean field ratings of B14A and N551 were 3.4 and 1.0, respectively. B14A and N551 both belong to the stiff stalk heterotic group (Figure 1b) and are related to one another as B14 was a founder of the synthetic population from which N551 was derived (Russell 2006). The IBS, as calculated from SNPs, between N551 and B14A was 0.82 which was greater than the average IBS of 0.67 in the diversity panel.

To perform inoculations, seeds of the inbred lines were planted in plastic inserts and placed in a greenhouse for two weeks before inoculations. Inoculations were carried out when the plants were at the V2 stage (Abendroth *et al.* 2011). For inoculations, plants were transported to the lab and were inoculated using a vacuum infiltration method with the Welch 1400 Duo Seal Vacuum Pump. Inoculum was prepared from *Cmn* isolate 12038 from the Alfano Lab (University of Nebraska-Lincoln), which was tested and determined to be virulent on maize. The bacterial cells were suspended in 10mM MgCl_2_ for measuring concentration and then were mixed into distilled water. The inoculum bacteria concentration was set to 1 × 10^8^ colony forming units with a spectrophotometer. To increase the surface tension of the suspension, 0.005% Tween20 was added to the inoculum. Control inoculations were done with water in place of the *Cmn* inoculum and samples were also collected from these controls at each time point. The plants were placed upside down into the inoculum and the vacuum was applied to each plant for three minutes.

Leaf samples were collected at 0, 8, and 15 hr post inoculations (hpi). All above ground leaves were cut with sterilized scissors and immediately placed in liquid nitrogen after wrapping in aluminum foil. These time points were chosen to evaluate genes that change expression patterns early in response to *Cmn* infection and contribute to primary defense response. In a preliminary RT-PCR experiment designed to determine optimal hpi, the pathogen responsive genes PR1 and PR5 were expressed within 12 to 15 hr. A previous study in Arabidopsis found that genes involved in early defense signaling responded to elicitor flg22 as early as 30 min after treatment (Asai *et al.* 2002).

Three biological replicates, with one plant representing a replicate, were included for each line, treatment, and hpi combination, resulting in a total of 30 samples (Supplementary Table 1). RNA was isolated using the Qiagen RNeasy mini kit (Qiagen, Valencia, CA, USA) based on the manufacturer’s protocol, and was purified using the Qiagen RNA clean-up protocol according to the manufacturer instructions.

### Library preparation and sequencing

RNA samples were submitted to the University of Minnesota Genomics Center for library preparation and sequencing. RNA sizing, quantification, and purity assessments were done with an Agilent Bioanalyzer (Agilent, Santa Clara, CA). Standard 36 dual indexed TruSeq RNA libraries were created. Libraries were sequenced on the Illumina HiSeq 2500 instrument using v4 chemistry (Illumina, Inc., San Diego, CA). Single end 50 bp reads were generated from the sequencing runs.

### Sequence quality control and transcript abundance estimates

Read quality was determined using FastQC version 0.11.5 (https://www.bioinformatics.babraham.ac.uk/projects/fastqc/). High contents of Illumina Universal and TruSeq adapters were detected and these adapters were removed with CutAdapt version 1.8.1 (Martin 2011). The quality cutoff value was set to 20 and a minimum processed read length value of 20 was used in CutAdapt while processing the reads. After adapter trimming, the reads were aligned to the B73 v4 reference genome using a splice-site aware aligner TopHat2 version 2.0.13 (Kim *et al.* 2013) with a minimum intron size (-i) of 5 and a maximum intron size (-I) of 60000. The B73 v4 genome assembly was downloaded from Gramene Release 33 (http://www.gramene.org). Read counting was performed with HTSeq version 0.5.3 (Anders *et al.* 2015**)** with strand specific option (-s) set to no, the feature type (-t) set to gene, the mode for handling overlapping reads (-m) set to union, and a minimum alignment quality (-a) of 20.

### Gene co-expression network anaylsis

The raw expression matrix of 39,324 genes was filtered to remove genes with consistently no counts across the samples. The filtered count matrix was read into the DESeq2 R package and log2(x+1) transformed (Love *et al.* 2014). A coefficient of variation (CV) filter was applied to filter out the genes with consistently low expression across the samples in which the genes with CV less than 0.5 were removed. After applying the transformation and CV filter, the expression matrix with 9,303 genes comprisong 26 samples out of 30 sequenced, was processed with R package WGCNA to identify gene co-expression modules (Langfelder and Horvath 2008). The blockwiseConsensusModules function from the WGCNA package was used to identify modules with tree cut height 0.4 and all other options set to default. WGCNA is a systems biology approach which uses the global gene expression matrix for all the genes to group the genes that show similar expression pattern together into co-expression ‘modules’.

Module eigen genes that were representative of the expression profile of all the genes inside each module were visualized by a heat map to find trends in expression profiles across the control and treated samples. The normalized and transformed expression matrix was centered and standardized to calculate the Z-scores and the expression profile of all the genes inside each module across the control and treated samples was plotted using parallel coordinate plots. A gene ontology (GO) analysis was performed using the AgriGO server to test if any of the modules were enriched in functions related to defense (Du *et al.* 2010).

It was hypothesized that the gene modules responding to inoculation with *Cmn* would explain a disproportionate amount of variation in WMD as compared to a bootstrapped set of random genes. GBS SNPs from the GWAS panel that fell inside genes of such module(s) and those that flanked within one kb (determined based on genome-wide average LD decay) were used to calculate a GRM, according to [3], and the proportion of phenotypic variation in Goss’s wilt resistance explained was calculated using a variance component approach (Gusev *et al.* 2014). To establish a baseline for determining whether the genes implicated by the gene module(s) explain a disproportionate amount of phenotypic variance, variance component approach was applied to 1,000 random sets of an equal number of genes as in the module of interest. It is important to note that the sampling unit is the gene, and all SNPs inside each sampled gene were used to calculate the GRM, meaning that the number of SNPs could vary across gene samples. To compare this approach to an approach where the number of SNPs was well held constant rather than genes, 1000 random sets of 83 SNPs were also sampled and used to generate a null distribution of variation explained using the variance component approach.

### Data availability

Phenotypic data used in this study is available at figshare GSA portal. FileS1 contains raw phenotypic data collected from 555 diversity panel lines, FileS2 contains Goss’s wilt BLUEs for diversity panel inbred lines, FileS3 contains subpopulation memberships (population structure) of 555 diversity panel lines computed using ADMIXTURE, FileS4 is contains raw phenotypic data for biparental populations, FileS5 contains Goss’s wilt BLUEs (1005 lines) used in Combined GWAS model, FileS6 contains membership assignments or population structure information used in Combined GWAS model, and FileS7 contains gene expression data used for WGCNA. Genotypic data used in this study was obtained from https://www.panzea.org/genotypes. Data are now available at CyVerse Data Store. CyVerse data store file path is /iplant/home/shared/panzea/genotypes/GBS/v27/. Supplemental material available at FigShare: https://doi.org/10.25387/g3.7884779.

## Results

### Significant pnenotypic variability for resistance to Goss’s wilt exists among inbred lines

A diverse panel of 555 maize inbred lines was screened for Goss’s wilt resistance over two years, forming the largest and most diverse set of lines screened for resistance to this gram positive bacterial disease to date. Variation in flowering time was restricted to reduce the confounding effects of flowering time on resistance (see Materials and Methods). Resistance scores for each plot were taken at multiple time points, which were then summarized by calculating the “weighted mean disease” (WMD). A skewed distribution toward resistance was observed (Figure 1a), similar to what was observed in an earlier evaluation of bi-parental mapping populations (Singh *et al.* 2016). Leaf blight symptoms were common following inoculation, while only a few plots showed severe wilting. In an analysis of variance on the diversity panel experiment, inbred line and inbred line-by-year interaction effects were found to be significant (*P* < 0.05). Broad-sense heritability on a plot basis was high (*H*^2^ = 0.75), indicating a combination of measurement precision and a large amount of genetic variation for Goss’s wilt WMD in this panel.

### Genetically similar lines vary in their level of resistance to Goss’s wilt

After filtering on the basis of SNP quality and minor allele frequency, a total of 342,237 SNP calls on the diversity panel remained. Linkage disequilibrium between SNPs was found to rapidly decay to 0.2 within 1000 bp, suggesting a high amount of diversity in the chosen panel. Principal component analysis and ADMIXTURE analysis (K = 3) using the SNP dataset revealed subpopulations within the diversity panel (Supplementary Figure 2). The first principal component (PC1) separated lines according to stiff stalk, non-stiff, and popcorn subpopulations. A few sweet corn and tropical lines included in the panel also clustered among themselves (Supplementary Figure 2a).

A neighbor joining tree of the diversity panel lines was created to visualize the subpopulations and distribution of Goss’s wilt resistance by subpopulation. Color coding of lines by subpopulation clearly indicated that the lines clustered according to subpopulation, as expected (Figure 1b). No strong pattern in the distribution of lines according to Goss’s wilt resistance score was observed (Figure 1c) as groups of highly related lines included both those that were resistant and susceptible. Although elite popcorn breeding lines are generally highly susceptible to Goss’s wilt (Rocheford *et al.* 1985), the popcorn lines screened as part of this diversity panel ranged from resistant to highly susceptible, indicating a high degree of variation even within popcorn (Figure 1c). It was found that lines closely related to Mo17 were generally resistant, and most of the lines related to B14 were moderately to highly susceptible, in agreement with previous findings (Calub *et al.* 1974).

### Genetic variation in Goss’s wilt resistance is controlled by many loci of small effect

**Figure 2:**
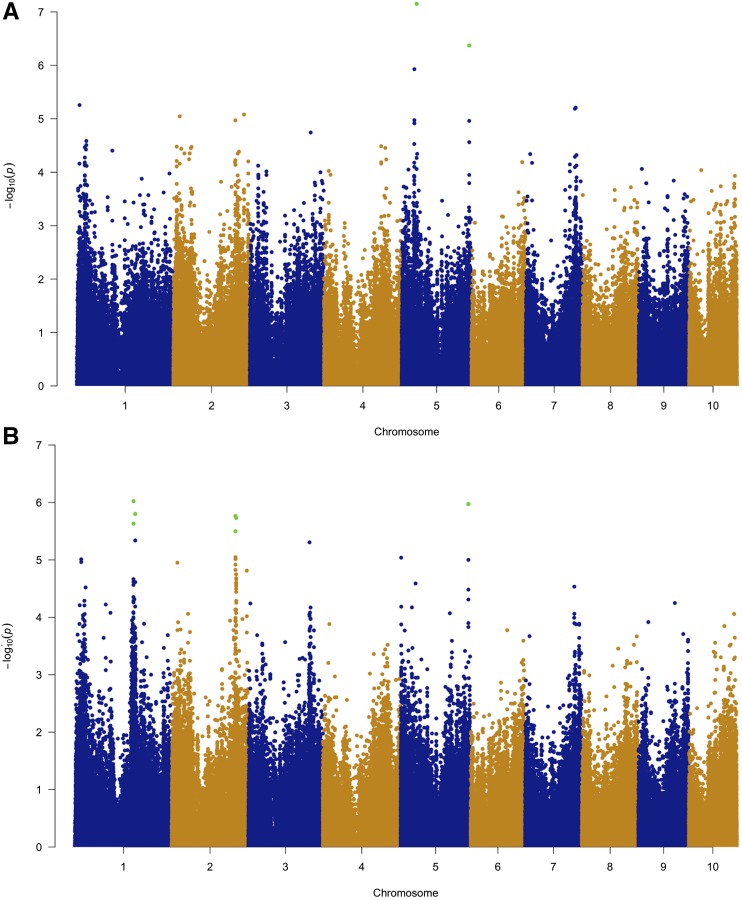
Manhattan plots showing results from genome-wide association mapping. (a) GWAS results on the diversity panel (N =555) displayed in a Manhattan plot where the y-axis is the negative log_10_ of p-values for the SNPs from model [2]. Associations that passed the false-discovery rate of 0.10 are colored green; (b) genome-wide association mapping results on the combined dataset (N =1005) displayed in a Manhattan plot where the y-axis is the -log_10_(P) for the SNPs from model [2]. Associations that passed the false-discovery rate of 0.10 are colored green.

Association analysis within the diversity panel identified three SNPs on chromosome 5 that passed a FDR cutoff of 0.10, with two of the SNPs being within just 17 bp of each other (Table 1, Figure 2a). The amount of phenotypic variation for Goss’s wilt resistance explained by individual SNPs within the diversity panel was 3–5% (Table 1). Together the three significant SNPs explained 8% of the phenotypic variation after accounting for population structure. In contrast, the polygenic genetic variance, modeled by the genomic relationship matrix (GRM) calculated using all SNPs, explained 64% of the phenotypic variation for Goss’s wilt resistance within the diversity panel.

**Table 1:** Information about significant SNPs associated with Goss’s wilt resistance in the diversity panel and combined dataset

Chrom†	Physical Positon of SNP (bp)‡	*P*-value§§	q-value¶	R^2^#	SNP effect¥	Candidate Gene£	Gene Function‡‡
Diversity panel							
5	46,455,199	7.01 x 10^−8^	0.02	0.05	0.28	GRMZM2G057459	Glutamate receptor
5	210,554,445	4.25 x 10^−7^	0.10	0.03	0.26	GRMZM2G368206	PHD finger domain, Zinc ion binding
5	210,554,466	4.25 x 10^−7^	0.10	—	0.26		
Combined dataset (diversity panel and bi-parental families)							
1	182,307,976	9.49 x 10^−7^	0.09	0.02	0.23	NA	
1	182,307,992	2.34 x 10^−6^	0.08	0.02	0.22	NA	
1	187,675,076	1.59 x 10^−6^	0.09	0.01	0.20	GRMZM2G132704	Nucleotide/RNA binding
						GRMZM2G132607	Ribokinase activity
						GRMZM2G132623	Constituent of ribosome
2	198,101,869	1.72 x 10^−6^	0.09	0.01	0.20	GRMZM2G048582	Response to Nitrogen
						GRMZM2G048551	Zinc ion binding
						GRMZM2G512469	Unknown
2	198,101,827	3.18 x 10^−6^	0.09	0.01	0.20		
2	198,101,829	3.18 x 10^−6^	0.09	—	0.20		
2	198,101,830	3.18 x 10^−6^	0.09	—	0.20		
2	200,227,875	1.86 x 10^−6^	0.09	0.01	0.21		
5	210,554,445	1.07 x 10^−6^	0.09	0.03	0.19	GRMZM2G368206	Protein binding, zinc ion binding
5	210,554,466	1.07 x 10^−6^	0.09	—	0.19		

† Chromosome.

‡ Physical position of the SNPs in base pairs.

§ P-value of the SNPs associated with Goss’s wilt from GWAS.

¶ False discovery rate or q-value of the SNPs, calculated from p-value.

# Variance explained by each SNP (R^2^)

¥ Additive effect of the SNP from GWAS.

£ Potential candidate genes in the region of significant SNPs.

‡‡ Annotated function of the potential candidate gene.

**Figure 3:**
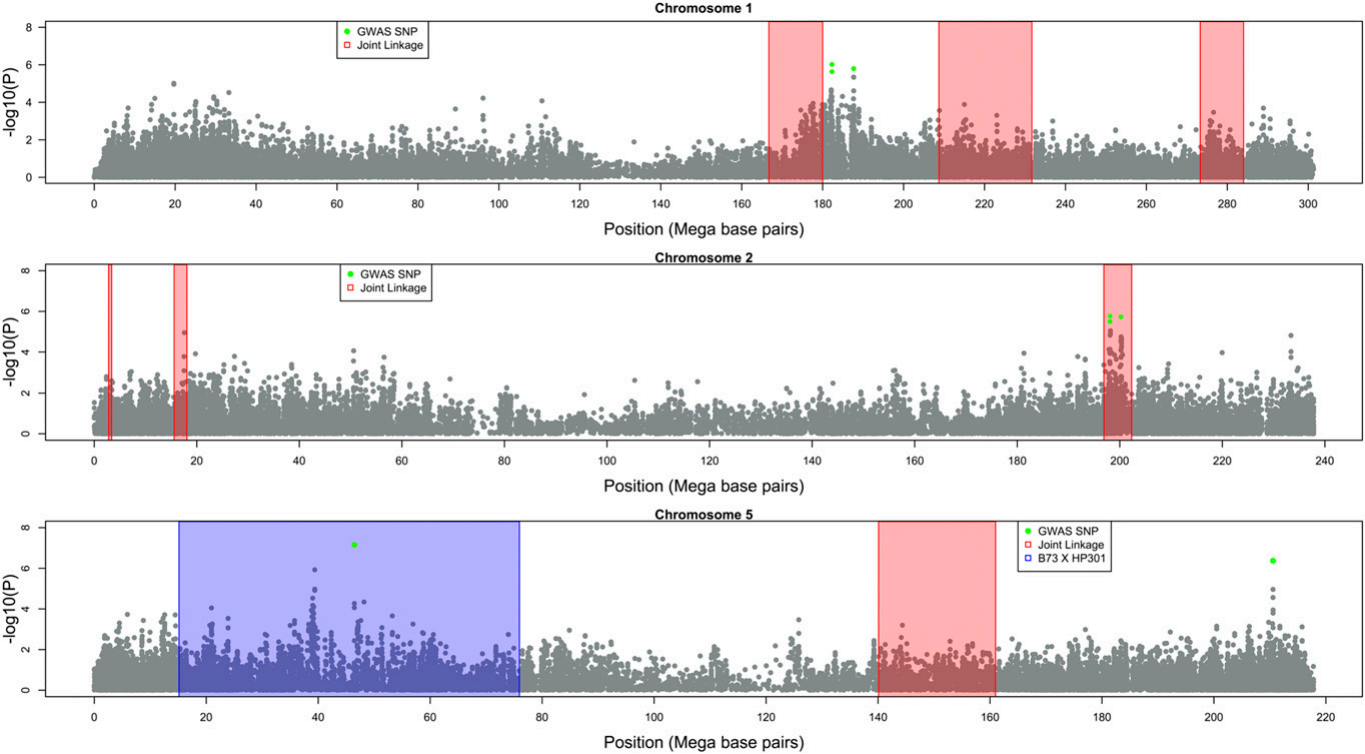
Comparison of physical positions of QTL detected in bi-parental linkage mapping and GWAS. Bi-parental linkage mapping was conducted by Singh *et al.* (2016), and significant SNPs from GWAS in the combined dataset co-located on chromosomes 1, 2, and 5. The x-axis of each plot represents the physical position of each SNP, and the y-axis displays the negative log_10_ of p-values for each SNP included in the GWAS. Gray colored solid points represent all SNPs used in GWAS. Significant SNPs in the GWAS are indicated by green dots, and 2-LOD support interval of QTL detected by Singh *et al.* (2016) are shown by the red or blue windows.

We subsequently conducted GWAS using a combined dataset that included both the diversity panel and three RIL populations. The phenotypic data for the three RIL populations was the same as that analyzed by Singh *et al.* (2016). Ten SNPs were found to be significant at a FDR of 0.10 in this analysis; these SNPs were on chromosomes 1, 2, and 5 (Table 1, Figure 2b). Each of the SNPs identified in the combined dataset explained a small amount of phenotypic variation for Goss’s wilt resistance, ranging from 1 to 3%. Physical positions of the significant SNPs found in GWAS of the combined dataset were compared to the QTL intervals of QTL mapping conducted using only the bi-parental families with fewer SNPs by Singh *et al.* (2016). Significant SNPs in GWAS on chromosome 2 co-localized with QTL peak from joint-linkage mapping and on chromosome 5 co-localized with QTL peak of the B73 x HP301 bi-parental population reported by Singh *et al.* (2016) (Figure 3). However, certain QTL were not detected, including QTL detected in both joint linkage mapping and within individual families (*e.g.*, chromosome 4, 9, and 10). Other QTL specific to one bi-parental family were not detected in GWAS as well (*e.g.*, chromosome 6, and 7).

Identification of candidate genes could contribute to an enhanced understanding of the biology underlying resistance to Goss’s wilt and provide a starting point for future gene confirmation studies. A candidate gene analysis was performed by extending a window around a given significant SNP until the average LD decayed to 0.20 on both sides of the significant SNP. If the defined window around the significant SNPs overlapped with the position of a gene, the gene was declared as a potential candidate. Several protein coding genes were identified within these windows with notable functions such as nucleic acid binding, zinc ion binding, electron transport, and a glutamate receptor protein (Table 1). In each of the regions with significant SNPs, the number of candidate genes ranged from zero to three (Table 1). One candidate gene on chromosome 5 was found to have a function directly related to plant defense. This gene codes for glutamate receptor protein and is a member of the glutamate receptor-like gene family (*GRLs*) that has been reported to play a role in plant defense response (Forde and Roberts 2014). Other candidate genes did not have an apparent direct role in defense to pathogens (Table 1).

### Haplotype block analysis of Goss’s wilt resistance QTL

A haplotype block analysis was conducted on each genomic region harboring significant SNPs to assess local LD decay rate and distribution of haplotype alleles among subpopulations. The haplotype blocks at the chromosome 1 and 5 regions were smaller in size due to low LD in these regions, while chromosome 2 had larger haplotype blocks (Supplementary Figure 3). Four of the five significant SNPs on chromosome 2 were located in block 1 (5 kb), while the fifth SNP (1,977,875 bp away) was excluded from the haplotype block analysis in this region (Supplementary Figure 3b). The chromosome 2 region was investigated further to look at the haplotype allele frequency in the diversity panel as a whole as well as within individual subpopulations. Multiple alleles were observed at each haplotype block within the region of interest on chromosome 2. The allelic effects were small, as the WMD of the lines carrying each of the haplotype allele ranged from 2.3 to 3.0 (Table 2). Allele A1 had the lowest mean Goss’s wilt WMD, and it was the most common allele in all subpopulations except for popcorn in which it had a frequency of only 0.02. Allele A2 had a higher Goss’s wilt WMD and a high frequency among the popcorn lines (93.8% of lines). The allele with the highest Goss’s wilt WMD, A3, had a relatively high frequency in the stiff stalk (29% of lines) and non-stiff stalk (20% of lines) subpopulations compared to the popcorn subpopulation (4.2% of lines), indicating there may be an allele carried by many stiff stalk and non-stiff stalk inbred lines that confers higher susceptibility than the allele common in the popcorn subpopulation (Table 2).

**Table 2:** Haplotype allele frequency in the diversity panel as a whole, and within individual subpopulations (stiff stalk, non-stiff stalk, popcorn, and unclassified). Haplotype blocks 1-4 are those at the chromosome 2 QTL region detected in the combined analysis of the diversity panel and RILs as shown in supplementary figure 2. Mean weighted mean disease (WMD) of the lines carrying each haplotype allele are presented

Block†	Haplotype Allele‡	Allele No.§	Haplotype Allele Frequency¶					Mean WMD¥
			Panel	Stiff	Non-stiff	Popcorn	Unclassified	
**BLOCK1 (5 kb)**	ATGG	A1	0.65	0.66	0.80	0.02	0.72	2.38
	AGGT	A2	0.17	0.05	0.01	0.94	0.10	2.73
	CGAG	A3	0.18	0.29	0.20	0.04	0.18	2.89
**BLOCK2 (79 kb)**	CCTCAGAAACCACGGCGGA	B1	0.38	0.47	0.44	0.00	0.47	2.35
	TTCTGGGATATAAAGCCTA	B2	0.09	0.01	0.01	0.47	0.07	2.85
	TTCTGAGATCCGCGCCGTA	B3	0.16	0.28	0.18	0.04	0.17	2.92
	CCTCAGATACCACGGCGTC	B4	0.11	0.06	0.22	0.00	0.14	2.47
	CCTCAGATACCACGGTGTC	B5	0.10	0.12	0.16	0.00	0.10	2.28
	TTTCGGGATCCGCGGCGTA	B6	0.08	0.05	0.00	0.49	0.06	2.57
**BLOCK3 (53 kb)**	GCC	C1	0.46	0.51	0.30	0.48	0.50	2.44
	ACC	C2	0.18	0.28	0.18	0.04	0.17	2.97
	GTT	C3	0.36	0.21	0.52	0.48	0.33	2.44
**BLOCK4 (13 kb)**	CGGACG	D1	0.57	0.81	0.53	0.04	0.63	2.53
	TATACG	D2	0.11	0.08	0.14	0.11	0.10	2.64
	TATACC	D3	0.23	0.04	0.31	0.37	0.22	2.56
	TATGGG	D4	0.10	0.07	0.03	0.48	0.05	2.50

† Haplotype block defined using four gamete rule implemented in software Haploview.

‡ An allele of a multiallelic haplotype.

§ Designation for multiple alleles of a haplotype.

¶ Frequency of each allele of a haplotype in the whole panel, stiff stalk, non-stiff stalk, popcorn, and unclassified sub-populations of the diversity panel.

¥ Mean WMD of the lines carrying each haplotype alleles.

### A module of co-expressed genes showed responsiveness to inoculations with CMN

**Figure 4:**
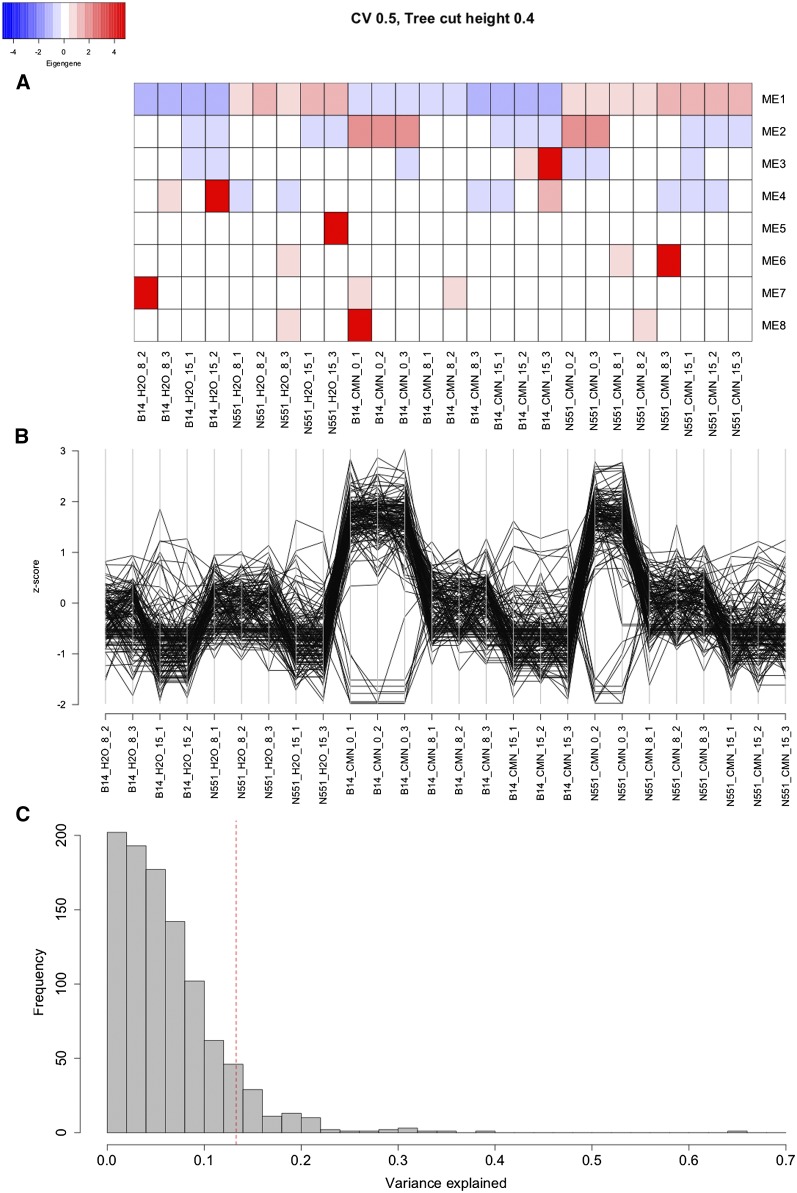
Expression patterns of eight modules identified using weighted gene co-expression network analysis (WGCNA) across different samples. (a) Heatmap of eigen genes of eight modules. An eigen gene is representative of the gene expression pattern of genes inside that module. Labels of the columns are inbred line_treatment_hours_rep; (b) Normalized expression of genes inside module two obtained from WGCNA across all the samples. This module showed changes in gene expression in both susceptible line B14A as well as resistant line N551 in response to *Cmn*; (c) Distribution of phenotypic variance explained by SNPs inside and flanking the 1,000 random samples of genes. As a comparison, phenotypic variation explained by SNPs inside and flanking the genes of module two is depicted by red vertical dashed line.

In addition to allelic variation, expression variation can contribute significantly to trait variation. To identify genes with expression variation that may be contributing to resistance to Goss’s wilt, a time course RNA sequencing experiment of a relatively resistant inbred line (N551) and susceptible inbred line (B14A) under control and infected conditions was performed. These lines were chosen for this experiment because they showed different levels of resistance to Goss’s wilt, and they are highly related to one another, reducing confounding genetic background effects (Figures 1b and 1c). Weighted gene co-expression network analysis identified eight modules of co-expressed genes in the time course experiment. The number of genes included in each module ranged from 30 in module eight to 1,557 in module 1, with 7,373 genes not clustering with any other genes. A heatmap of the eigen genes of the modules provides a high-level overview of each module, allowing identification of trends in gene expression changes of possible biological importance. We did not find any modules showing changes in gene expression in response to infection that were specific to a genotype (Figure 4a). However, module two showed changes in gene expression in both lines in response to *Cmn* (Figure 4a). In both N551 and B14A, the 141 genes that clustered into module two showed a decrease in expression at eight hpi, and an even further decrease in expression at 15 hpi (Figure 4a). Further evaluation of genes inside the second module from parallel coordinate plots revealed that most genes were down-regulated in B14A and N551 at eight and 15 hpi with *Cmn* (Figure 4b). Module one was genotype specific module and rest of the modules appear to indicate variation at the sample level.

A gene ontology (GO) enrichment analysis revealed that the genes of the second module were not enriched for defense functions. Most of the significant Biological Process related GO terms pointed toward regulation of metabolism (Supplementary Table 2). Checking the functional annotations of genes inside the second module revealed several protein coding genes including protease inhibitor, wound induced proteins, Thiazole biosynthetic enzyme, aquaporin, DRE-binding protein, etc. (Supplementary Table 3).

We wanted to test whether SNPs within and flanking (up to one kb on either side) the genes in module two explained a disproportionate amount of the variance in Goss’s wilt resistance observed in the diversity panel to help determine if these genes are important contributors to genetic variance for quantitative disease resistance. The physical positions of the SNPs used in the GWAS were aligned with the physical positions of the genes that clustered into module two. Twenty genes in module two had at least one SNP within or flanking them, with the remaining 121 genes having no SNPs within or flanking them. The total number of SNPs found was 83. These 83 SNPs were used to calculate a GRM, which was then fit into a linear mixed model to estimate the variance in WMD explained by these SNPs (Yang *et al.* 2010; Gusev *et al.* 2014). The SNPs of module two explained 13.3% of the phenotypic variation for WMD, which is higher than the total percent variation explained by the significant GWAS SNPs. To determine if the variance explained by the SNPs of genes included in module two was greater than expected by a random set of genes, additional GRMs were calculated using SNPs inside and flanking 1,000 random samples of 20 genes. The number of SNPs in the 1,000 random samples ranged from 0 to 199 with a mean of 39 SNPs. The percent variation explained by the SNPs among the 1,000 random gene samples ranged from 0.0 to 64.8%. Only 8.6% of the 1,000 random samples explained more variation than the module two SNPs (Figure 4c), providing suggestive, but not strong nor statistically significant evidence for a role of these genes in Goss’s wilt resistance at the field level. A similar analysis was performed where 1000 random sets of SNPs were sampled in which the number of SNPs inside and flanking genes (83) was held constant rather than the number of genes. The variation explained by these 1000 sets of SNPs ranged from 5 to 20%, with 9.4% of the random samples explaining more variation than the 83 SNPs implicated by the gene expression analysis. These results led to the same conclusion as the results obtained where the gene was considered as the sampling unit.

## Discussion

Genome-wide association mapping, made possible by the widest screening of Goss’s wilt resistance reported to-date, and gene co-expression network analysis were used to increase our knowledge of the genetic basis of resistance to Goss’s wilt of maize. Goss’s wilt is an important disease of maize that has not been studied extensively using modern technologies of molecular genetics as compared to other maize diseases such as NCLB, SCLB, and gray leaf spot (Kump *et al.* 2011; Poland *et al.* 2011; Benson *et al.* 2015), only three published studies have used molecular markers to study the genetic basis of Goss’s wilt resistance (Schaefer and Bernardo 2013; Singh *et al.* 2016; Cooper *et al.* 2018). Both GWAS and gene co-expression network analysis approaches pointed toward a complex nature of resistance to Goss’s wilt, which is in agreement with the complex nature of plant signaling and defense processes as studied using model organisms (Jones and Dangl 2006; Bonardi and Dangl 2012) and hypothesized to be the case for Goss’s wilt based on initial genetic studies (Martin *et al.* 1975; Rocheford *et al.* 1989; Treat *et al.* 1990).

A great degree of variation for Goss’s wilt resistance was found within subpopulations, with relatively little variation between subpopulations in most cases. Our expectation was that inbred lines within certain subpopulations would be more resistant on average as compared to inbred lines from other subpopulations. This was, however, not observed in this study as resistant and susceptible lines were distributed among all the groups (Figure 1c). The popcorn subpopulation was more susceptible overall, but relatively resistant popcorn inbred lines were still found. Lines related to inbred line B14 tended to be susceptible whereas the lines related to Mo17 were found to be generally resistant, as has been reported in previous studies (Schuster *et al.* 1972; Calub *et al.* 1974).

The GWAS and calculated proportion of variance explained by all genotyped SNPs points toward a highly polygenic genetic architecture underlying Goss’s wilt resistance. Similar to our previous QTL mapping study (Singh *et al.* 2016), each of the significant SNPs associated with Goss’s wilt resistance in the GWAS explained a small amount of the phenotypic variation. For other important leaf diseases of maize including southern corn leaf blight, northern corn leaf blight, and gray leaf spot several small effect SNPs have been associated with resistance (Kump *et al.* 2011; Poland *et al.* 2011; Benson *et al.* 2015). Thirty-two QTL with small additive effects that together explained 80% and 93% of the phenotypic and genotypic variation, respectively, for southern corn leaf blight were reported in a GWAS of maize NAM population (Kump *et al.* 2011). Similarly, 29 QTL were identified for northern corn leaf blight in the NAM population that explained 77% and 96% of the phenotypic and genetic variance, respectively, for northern corn leaf blight, with each QTL being of small effect (Poland *et al.* 2011). The studies using the NAM population explained greater total phenotypic variation because of the nested linkage mapping design of the NAM population combined with a much larger population size. Nevertheless, the polygenic variance accounted for 64% of the phenotypic variation for Goss’s wilt resistance in the diversity panel and thus a genomic selection approach may be more effective in breeding for Goss’s wilt resistance compared to selecting for individual QTL. A similar conclusion was reported in a *Fusarium* ear rot study in maize in which the GRM explained nearly 50% of the variation for *Fusarium* ear rot and only 1.3–3% of the variation was explained by individual significant SNPs (Zila *et al.* 2013).

Combined GWAS (diversity panel and bi-parental populations together) increased the power of GWAS and more genomic regions were associated with Goss’s wilt resistance as compared to GWAS of the diversity panel only. However, there were some QTL regions that were detected in joint-linkage and linkage mapping of individual bi-parental populations conducted by Singh *et al.* (2016) that were not detected by combined GWAS. One possible cause of not detecting certain QTL in combined GWAS approach could be different predominant linkage phases between SNPs and causal polymorphisms between bi-parental populations and the diversity panel, possibly causing effects to be cancelled out. Also, because the number of markers was much larger for GWAS, the higher statistical threshold of GWAS needed to keep the experiment-wise false positive error rate in check likely resulted in failure to declare significance for smaller effect QTL.

In addition to the variation at the allelic level, differential regulation of genes in response to pathogen may contribute to the phenotypic variation. Furthermore, the number of host genes regulated by disease resistance is a basic systems biology question that has been explored multiple times with different plant pathosystems. In a gene-co-expression network analysis of response of Citrus to bacterium *Candidatus liberibacter* spp., 3,507 genes were suggested to play a role in defense (Zheng and Zhao 2013). Similarly, gene co-expression network analysis of genes regulated by immune and hypersensitive resistance responses to *Blumeria graminis* f. sp *tritici* in diploid wheat *Triticum urartu* indicated that 3,900 and 4,100 genes may be involved in the above two types of resistance responses, respectively (Zhang *et al.* 2016). In the present study, we identified one module that corresponded to response of maize inbred lines to *Cmn* were identified consisting of 141 co-related genes. Most of the genes in this module showed decreased expression in susceptible and resistant inbred lines at at 8 and 15 hpi. This result corroborates with previous findings from molecular and co-expression network studies showing that plant immunity can be controlled by negative regulation of a certain set of genes (Sato *et al.* 2010; Segonzac *et al.* 2014). Particularly, specific subunits of protein Ser/Thr phosphatases have been involved in negative regulation of defense signaling at different steps (Segonzac *et al.* 2014). Negative regulation of certain genes within a plant immune system are required for effective functioning by preventing over activation of the immune system which may cause auto immune responses such as cell death, thus reducing plant fitness (Sato *et al.* 2010).

A search for overlap between genes implicated by the WGCNA and variation explained in Goss’s resistance at the field level was performed to determine the extent to which genes in module two are important to variation in resistance among inbred lines. None of the eight candidate genes identified in the GWAS analysis overlapped with the genes inside of module two. Several reasons could explain this lack of strong correspondence between GWAS and WGCNA. The power of the GWAS was low such that it could not be expected to detect all the phenotypic variation due to possible low frequencies of the causal alleles and rapid LD decay in this panel. Second, failure of WGCNA implicated genes to explain more variation in Goss's wilt resistance than random samples of genes could be because the variation in gene expression of these genes is not related to polymorphisms within the genes, but rather to polymorphisms in *cis*- or *trans*-acting transcription factors. Third, it is possible that the gene expression differences between the two chosen inbred lines, B14A and N551, are not representative of the gene expression patterns in the diversity panel as a whole. Fourthly, the genes contributing to quantitative disease resistance in the field, 15 days or more after inoculation may not be the same, or have little overlap with, genes responding to infection within hours during the seedling stages. Finally, while a mixture of five isolates was used for field inoculations of the GWAS, only one isolate was used to inoculate B14A and N551 in the lab.

Using a variance component approach, we found that the genes implicated in the WGCNA did not explain a disproportionate amount of the variance in Goss’s wilt resistance in the diversity panel using a significance level of *P* < 0.05. However, only 8% of 1,000 random genes samples explained more phenotypic variance than those genes implicated by the WGCNA, suggesting a possible role of these genes. More research is needed to precisely determine the role of these genes in quantitative Goss’s resistance at the field level. More generally, these results suggest a possible useful role for combining WGCNA with quantitative genetic analyses to determine genes underlying variation for complex disease resistance traits in crops. More research to refine these methods to attain this overall goal is warranted.

Goss’s wilt and leaf blight of maize is an economically important disease, yet very few studies have been performed with the aim of elucidating the genetic basis of host resistance using modern genomic and transcriptomic techniques. This study explored an extensive amount of genetic variation for Goss’s wilt resistance and identified SNPs associated with resistance using a GWAS approach. The amount of variation attributed to the background polygenic effect through a genomic relationship matrix was eightfold higher than that explained by statistically significant QTL alone. These results suggest the genetic architecture of this disease is highly polygenic, which is consistent with the findings of our previous linkage mapping study and those using classical mating designs. Several biological processes may be involved in quantitative resistance to Goss’s wilt which were revealed by GO analysis and functional annotations of module two genes. Results from this study provide important information about the number of QTL, effect size of QTL, and the candidate genes underlying Goss’s wilt resistance, and provides a critical step toward further elucidating the genetic mechanisms of Goss’s wilt resistance. Due to the presence of several small effect QTL that together contribute to resistance to Goss’s wilt, a single QTL likely cannot be targeted for incorporating resistance into maize germplasm through marker-assisted selection. Rather, a genome-wide selection approach for population improvement maybe a better strategy for imparting resistance to Goss’s wilt into maize breeding programs.
